# Specific RNA structures in the 5′ untranslated region of the human cytomegalovirus major immediate early transcript are critical for efficient virus replication

**DOI:** 10.1128/mbio.02621-23

**Published:** 2024-01-02

**Authors:** Bekah Dickmander, Andrew Hale, Wes Sanders, Erik Lenarcic, Ben Ziehr, Nathaniel J. Moorman

**Affiliations:** 1 Department of Microbiology and Immunology, Lineberger Comprehensive Cancer Center, University of North Carolina at Chapel Hill, Chapel Hill, North Carolina, USA; University of Pennsylvania, Philadelphia, Pennsylvania, USA; University of Pennsylvania Perelman School of Medicine, Philadelphia, Pennsylvania, USA

**Keywords:** human herpesvirus, human cytomegalovirus, HCMV, mRNA translation, protein synthesis, major immediate early gene, IE1, IE2, lytic replication

## Abstract

Human cytomegalovirus (HCMV) requires the robust expression of two immediate early proteins, IE1 and IE2, immediately upon infection to suppress the antiviral response and promote viral gene expression. While transcriptional control of IE1 and IE2 has been extensively studied, the role of post-transcriptional regulation of IE1 and IE2 expression is relatively unexplored. We previously found that the shared major immediate early 5′ untranslated region (MIE 5′ UTR) of the mature IE1 and IE2 transcripts plays a critical role in facilitating the translation of the IE1 and IE2 mRNAs. As RNA secondary structure in 5′ UTRs can regulate mRNA translation efficiency, we used selective 2′-hydroxyl acylation analyzed by primer extension and mutational profiling (SHAPE-MaP) to identify RNA structures in the shared MIE 5′ UTR. We found that the MIE 5′ UTR contains three stable stem loop structures. Using a series of recombinant viruses to investigate the role of each stem loop in IE1 and IE2 protein synthesis, we found that the stem loop closest to the 5′ end of the MIE 5′ UTR (SL1) is both necessary and sufficient for efficient IE1 and IE2 mRNA translation and HCMV replication. The positive effect of SL1 on mRNA translation and virus replication was dependent on its location within the 5′ UTR. Surprisingly, a synthetic stem loop with the same free energy as SL1 in its native location also supported wild type levels of IE1 and IE2 mRNA translation and virus replication, suggesting that the presence of RNA structure at a specific location in the 5′ UTR, rather than the primary sequence of the RNA, is critical for efficient IE1 and IE2 protein synthesis. These data reveal a novel post-transcriptional regulatory mechanism controlling IE1 and IE2 expression and reinforce the critical role of RNA structure in regulating HCMV protein synthesis and replication.

**IMPORTANCE:**

These results reveal a new aspect of immediate early gene regulation controlled by non-coding RNA structures in viral mRNAs. Previous studies have largely focused on understanding viral gene expression at the level of transcriptional control. Our results show that a complete understanding of the control of viral gene expression must include an understanding of viral mRNA translation, which is driven in part by RNA structure(s) in the 5′ UTR of viral mRNAs. Our results illustrate the importance of these additional layers of regulation by defining specific 5′ UTR RNA structures regulating immediate early gene expression in the context of infection and identify important features of RNA structure that govern viral mRNA translation efficiency. These results may therefore broadly impact current thinking on how viral gene expression is regulated for human cytomegalovirus and other DNA viruses.

## INTRODUCTION

Human cytomegalovirus (HCMV) is a ubiquitous herpesvirus that establishes lifelong infection in the majority of the world’s population. While mainly asymptomatic in healthy individuals, HCMV infection can have serious consequences for immunocompromised patients and newborns. A primary HCMV infection during pregnancy is associated with an increase in congenital birth defects. Primary infection or reactivation of latent HCMV in immunocompromised adults such as transplant patients can lead to organ failure, leukopenia, and encephalitis (1–4). Thus, understanding the mechanisms by which HCMV infection leads to disease is important for developing new therapeutics for a variety of patient populations.

During lytic HCMV infection, genes are transcribed in a temporally regulated cascade divided into three kinetic classes: immediate-early, early, and late genes (4, 5). The expression of each class relies on the successful expression of the preceding class. As such, expression of HCMV immediate early genes is critical for successful HCMV replication. In particular, the HCMV IE1 and IE2 genes are expressed to high levels early in infection and play critical roles in establishing lytic replication by transactivating both host and viral gene expression (5). IE1 prevents the deposition of suppressive chromatin marks on the HCMV genome by regulating histone deacetylase activity and also inhibits type 1 interferon response (6–8). IE2 stimulates the expression of host genes (9–13) and viral genes (14–20) to promote HCMV replication. IE1 is required for efficient virus replication at a low multiplicity of infection, while deletion of the IE2 gene results in a complete block to viral replication (21), highlighting the importance of IE1 and 2 expression for HCMV replication.

IE1 and IE2 protein expression is regulated at multiple levels in a dynamic manner throughout infection. Several cellular (22–29) and viral (29–34) factors regulate IE1 and IE2 expression at the level of transcription. Post-transcriptional events also regulate IE1 and IE2 expression during HCMV infection. For example, HCMV uses cellular machinery to regulate IE1 and IE2 alternative splicing (35–39). Additionally, transcripts encoding IE1 and IE2 associate with polysomes in a dynamic manner throughout infection (40). Protein stability and degradation also play critical roles in regulating HCMV genes; IE1 is a metabolically stable protein with an estimated intracellular half-life between 21 and 30 hours (41, 42), whereas IE2 has a much shorter half-life of 2.5 hours in cell (41, 43). While the examples above show a critical role of post-transcriptional regulatory mechanisms in the control of IE1 and IE2 expression, there is a lack of understanding the mechanisms and factors regulating IE1 and IE2 expression at the post-transcriptional level.

A critical regulatory element in post-transcriptional regulation of gene expression is the 5′ untranslated region (5′ UTR) of an mRNA (44). Multiple 5′ UTR features, such as length, sequence composition, and secondary structure, impact mRNA translation (44–53). Early in infection mature IE1 and IE2 mRNAs are derived from a single primary transcript whose transcription is driven by the HCMV major immediate early promoter (MIEP) (5, 54). Alternative splicing gives rise to unique IE1 and IE2 transcripts, which differ in their 3′ terminal exon. However mature IE1 and IE2 transcripts arising from the MIEP share the same 5′ UTR (55), suggesting the potential for a common role for the shared IE1 and IE2 5′ UTR (MIE 5′ UTR) in regulating both IE1 and IE2 protein expression. We previously found that the wild type sequence of the MIE 5′ UTR is an important positive regulator of IE1 and IE2 mRNA translation and HCMV replication (56). While these data revealed the critical role for the MIE 5′ UTR in HCMV replication, the specific sequences and/or RNA structures important for IE1 and IE2 expression remain undefined.

To begin to understand how RNA sequences and structure in the MIE 5′ UTR affect IE1 and IE2 expression, we used selective 2′-hydroxyl acylation analyzed by primer extension and mutational profiling (SHAPE-MaP) to generate an experimentally validated model of the RNA secondary structure of the shared MIE 5′ UTR (57). Based on these data, we designed a series of recombinant HCMV strains containing mutations in specific RNA structures and measured the effect of each change on IE1 and IE2 mRNA translation and virus replication. We identified a specific RNA stem-loop in the MIE 5′ UTR, named SL1, that was both necessary and sufficient for efficient mRNA translation, and defined critical features of SL1 important for its activity. Together these data reveal that RNA structure plays a previously unappreciated role in regulating IE1 and IE2 gene expression and virus replication and suggest RNA structure may play similar roles in regulating the expression of additional viral genes.

## MATERIALS AND METHODS

### Cells and viruses

MRC5 human fibroblasts and HeLa cells were cultured at 37°C in Dulbecco’s modified Eagle medium (DMEM; Sigma) containing 10% fetal bovine serum (Gibco) and penicillin-streptomycin (Sigma). Virus stocks were grown on MRC5 fibroblasts and titers were determined via the 50% tissue culture infectious dose (TCID_50_) assay. AD*in*GFP (58) was used as the wild-type HCMV strain for all experiments.

Recombinant viruses were made as previously described (32, 55, 59, 60). Briefly, *Escherichia coli* (SW105) isolates containing the AD*in*GFP bacterial artificial chromosome (BAC) were incubated at 42°C for 15 minutes to become recombination competent. The bacteria were electroporated with a kanamycin-levansucrase (KanSacB) expression cassette with 50 nucleotides flanking the insertion site for recombination into the HCMV genome. The KanSacB template was generated by PCR using primers IE UTR del KS F and IE UTR del KS R, listed in Table 1. Following recombination, bacteria were selected on kanamycin plates. Colonies were screened for gross recombination by restriction digest. PCR amplification was used to determine the correct insertion of the KanSacB cassette, which was then confirmed via sequencing. A second round of recombination was used to replace the KanSacB cassette with the intended mutation. The full CAA recombinant was made as previously described (56), first by annealing oligonucleotides “CAA Oligo F” and “Full CAA Oligo R” before amplification using primers IE UTR CAA F and IE UTR CAA R (Table 1). The remaining recombinant viruses were made by amplifying the oligonucleotide containing the desired mutation with IE Mutant F and IE Mutant R. The PCR product for each recombinant was electroporated into recombination-competent KanSacB bacteria, and colonies were selected for growth on LB agar with 6% sucrose which is toxic in the presence of the SacB gene. Replica plating was used to confirm the absence of kanamycin resistance indicating successful removal of the kanamycin cassette, and colonies were screened for gross recombination via restriction digest. The +500 to −500 nucleotides surrounding the recombination site were PCR amplified using IE Mutant Seq F and IE Mutant Seq R and sequenced to confirm correct sequence insertion into the HCMV genome. BAC DNA was purified by Nucleobond and electroporated into MRC5 fibroblasts. Two independent isolates were generated and characterized for each recombinant virus from the wild-type BAC. Primer and oligonucleotide sequences can be found in Table 1.

**TABLE 1:** Sequence of primers and oligonucleotides used in this study^*a*^

Primer name	Sequence (5′–3′)
qRT-PCR GAPDH F	GTCTCCTCTG ACTTCAACAG CG
qRT-PCR GAPDH R	ACCACCCTGT TGCTGTAGCC AA
qRT-PCR IE1 F	CAAGTGACCG AGGATTGCAA
qRT-PCR IE1 R	CACCATGTCC ACTCGAACCT T
qRT-PCR IE2 F	TGACCGAGGA TTGCAACGA
qRT- PCR IE2 R	CGGCATGATT GACAGCCTG
qRT-PCR UL99 F	GTGTCCCATT CCCGACTCG
qRT-PCR UL99 R	TTCACAACGT CCACCCACC
IE Mutant Seq F	GGCTCATGTCC AACATTACCG C
IE Mutant Seq R	CCAAATGCGT CAGCGGTGCA AG
IE Mutant F	CGGTAGGCGT GTACGGTGGG AGGTCTATAT AAGCAGAGCT CGTTTAGTGA
IE Mutant R	AGCATGCATA AGAAGCCAAG GGGGTGGGCC TATAGACTCT ATAGGCGGTA
IE UTR CAA F	AGGCGTGTAC GGTGGGAGGT CTAT
IE UTR CAA R	CCAAGGGGGT GGGCCTATAG AC
IE UTR del KS F	AGGCGTGTAC GGTGGAGGTC TATATAAGCA GAGCTCGTTT AGTGAACCGG AATTCGAGCT CGGTACCCGG
IE UTR del KS R	AGCCAAGGGG GTGGGCCTAT AGACTCTATA GGCGGTACTT ACGTCACTCT TGATCCCGGG AAAAGTGCCA CC
+6	GCAGAGCTCG TTTAGTGAAC CGTCAACAAG CCTGGA**GACG** **CCA**TCCACGC TGTTTTGACC TCCA**TAGAAG** **A**CACCG**GGA**C CG**ATCCA**GCC **TCCGC**GGCCG G**GAA**CGGTG**C** **AT**TGGAACGC GG**ATTC**CCCG TGCCAAG**AGT** GACGTAAGTA CCGCCTATAG AGTCTA
SL23	GCAGAGCTCG TTTAGTGAAC CGTCAACAAC AACAACAACA ACAACAACAA CAACAACAAC TCCA**TAGAAG** **A**CACCG**GGA**C CG**ATCCA**GCC **TCCGC**GGCCG G**GAA**CGGTG**C** **AT**TGGACAAC AACAACAACG TGCCAAG**AGT** GACGTAAGTA CCGCCTATAG AGTCTA
SL1	GCAGAGCTCG TTTAGTGAAC CGTCAACAAG CCTGGA**GACG** **CCA**TCCACGC CAACAACAAC AACAACAACA ACAACAACAA CAACAACAAC AACAACAACA ACAACAACAA CAACAACAAC AACAACAACA ACAACAACAA CAAGTAAGTA CCGCCTATAG AGTCTA
SL2	GCAGAGCTCG TTTAGTGAAC CGTCAACAAC AACAACAACA ACAACAACAA CAACAACAAC TCCA**TAGAAG** **A**CACCG**GGA**C CG**ATCCA**GCC **TCCGC**GGCCG G**GAA**CGGTG**C** **AT**TGGACAAC AACAACAACA ACAACAACAA CAAGTAAGTA CCGCCTATAG AGTCTA
SL3	GCAGAGCTCG TTTAGTGAAC CGTCAACAAC AACAACAACA ACAACAACAA CAACAACAAC AACAACAACA ACAACAACAA CAACAACAAC AACAACAACA ACAACAACAA CAACAACAAC AACAACAACG TGCCAAG**AGT** GACGTAAGTA CCGCCTATAG AGTCTA
SL123	GCAGAGCTCG TTTAGTGAAC CGTCAACAAG CCTGGA**GACG** **CCA**TCCACGC CAACAACAAC TCCA**TAGAAG** **A**CACCG**GGA**C CG**ATCCA**GCC **TCCGC**GGCCG G**GAA**CGGTG**C** **AT**TGGACAAC AACAACAACG TGCCAAG**AGT** GACGTAAGTA CCGCCTATAG AGTCTA
SL1-1	GCAGAGCTCG TTTAGTGAAC CGTCAACAAG CCTGG**AAAAA** **AAA**TCCACGC CAACAACAAC AACAACAACA ACAACAACAA CAACAACAAC AACAACAACA ACAACAACAA CAACAACAAC AACAACAACA ACAACAACAA CAAGTAAGTA CCGCCTATAG AGTCTA
SL1-2	GCAGAGCTCG TTTAGTGAAC CGTCAACAAG AGCCTG**GACG** **CCA**CAGGGTC CAACAACAAC AACAACAACA ACAACAACAA CAACAACAAC AACAACAACA ACAACAACAA CAACAACAAC AACAACAACA ACAACAACAA CAAGTAAGTA CCGCCTATAG AGTCTA
SL1-3	GCAGAGCTCG TTTAGTGAAC CGTCAACAAG TGCAGC**GACG** **CCA**GCTGGAC CAACAACAAC AACAACAACA ACAACAACAA CAACAACAAC AACAACAACA ACAACAACAA CAACAACAAC AACAACAACA ACAACAACAA CAAGTAAGTA CCGCCTATAG AGTCTA
SL1-4	GCAGAGCTCG TTTAGTGAAC CGTCAACAACA ACAACAACAA CAACAACAAC AACAACAAC AAGCCTGGAG **ACGCCA**TCCA CGCCAACAAC AACAACAACA ACAACAACAA CAACAACAAC AACAACAACA ACAACAACAA CAAGTAAGTA CCGCCTATAG AGTCTA
CAA Oligo F	AGGCGTGTAC GGTGGGAGGT CTATATAAGC AGAGCTCGTT TAGTGAACCG CAACAACAAC AACAACAACA ACAACAACAA CAACAACAAC AACAACAACA ACAACAACAA CAACAACAAC AACAACAACA ACAACAACAA CAACAACAAC AACAACAACA ACCAAGAGTG ACG
CAA Oligo R	CCAAGGGGGT GGGCCTATAG ACTCTATAGG CGGTACTTAC GTCACTCTTG GTTGTTGTTG TTGTTGTTGT TGTTGTTGTT GTTGTTGTTG TTGTTGTTGT TGTTGTTGTT GTTGTTGTTG TTGTTGTTGT TGTTGTTGTT GTTGTTGTTG TTGTTGTTGT TGCGGTTCACT AAAC

^*a*^ SL1 is underlined, and loop sequences in all structures are shown in bold.

### SHAPE modification and library generation

RNA was folded for 15 minutes at 37°C with 10 mM MgCl_2_ and 111 mM KCl. 100 nM of 1-methyly-7-nitroisatoicanhydrde (1M7) was added to the modified RNA reaction, followed by a 5-minute incubation at 37°C. Unmodified RNA was treated with dimethyl sulfoxide (DMSO) prior to a 5-minute incubation at 37°C. The denatured RNA was incubated at 95°C for 2 minutes before the addition of 100 nM 1M7 and a 2-minute incubation at 95°C. All RNAs were purified using the RNA Clean and Concentrator −5 Kit (Zymo Research). Once purified, RNAs were random primed (Random Primer 9 NEB) and incubated at 65°C for 5 minutes followed by rapid cooling on ice. DTT (0.1 M), dNTPs (10 mM), Tris pH 8.0 (500 mM), KCl (750 mM), MnCL_2_ (500 mM), and Superscript II Reverse Transcriptase (Thermo Fisher Scientific) were added to the primed RNAs and incubated at 25°C for 15 minutes followed by a 3-hour incubation at 42°C. The reaction was stopped by incubating the reaction at 70°C for 15 minutes. Following reverse transcription, RNAs were cleaned using Illustra MicroSpin G-50 columns (GE Healthcare). Double-stranded DNA was made using NEBNext Ultra II Non-Directional RNA Second Strand Synthesis Module (NEB) and purified using PureLink PCR Micro Kit (Thermo Fisher Scientific). Double-stranded DNA was quantified using Qubit dsDNA HS Assay Kit (Thermo Fisher Scientific). Next-generation sequencing libraries were made using Nextera XT DNA Library Preparation Kit (Illumina) and cleaned using Agencourt AMPure XP (Beckman Coulter) before quantification using Qubit dsDNA HS Assay Kit. Libraries were sequenced on a MiSeq Desktop Sequencer (Illumina) using a MiSeq Reagent Kit v3 (600-cycle; Illumina). Data were analyzed using ShapeMapper v1.2 and SuperFold v1.0 and visualized on Varna v3-93.

### Luciferase assays

Gblocks (IDT) containing a T7 transcription promoter and the mutant IE1 UTR sequences were cloned into the pGL3 Control plasmid upstream of the firefly luciferase open reading frame using Gibson (NEB). mRNAs were transcribed from linearized plasmids using mMessage mMachine (ThermoFisher). Translation-competent cytosolic extracts were produced from uninfected or HCMV-infected fibroblasts at 6 hours post infection. 90% confluent cells were collected and resuspended in an equal volume of extraction buffer (10 mM HEPES, pH 7.6, 10 mM potassium acetate, 0.5 mM magnesium acetate, 5 mM DTT, and complete protease inhibitors [Roche]), incubated at 4°C for 45 minutes and then sheared through a 21 gauge needle 15 times. After insoluble material was removed by centrifugation, the extracts were diluted to have equal total protein concentrations, aliquoted, and frozen at −80°C. Endogenous mRNAs in each extract were removed by incubation at room temperature for 7 minutes after the addition of 1,500 U/mL micrococcal nuclease (NEB) and 0.75 mM CaCl_2_. Nuclease was then inactivated by the addition of 1.5 mM EGTA. Small scale *in vitro* translation reactions were assembled using cytosolic extract, 40 units RNasin (Promega), 125 mM potassium acetate, 1.6 mM HEPES, pH 7.6, 2 mM creatine phosphate, 10 ng/µL creatine kinase, 10 µM amino acids, and 150 ng *in vitro* transcribed firefly luciferase mRNA. Reactions were incubated at 37°C for 30 minutes and then mixed with luciferase assay reagent (Promega) before being analyzed for luciferase activity using a luminometer (Promega GloMax Navigator). Capped, *in vitro* synthesized RNA was transfected into HeLa cells using Lipofectamine 2000 (Invitrogen) according to the manufacturer’s protocol. At 4 hours post transfection, the media was removed, and the cells were washed with PBS. The cells were lysed in passive lysis buffer (Promega) for 15 minutes at room temperature before luciferase activity was measured using a luminometer as described above.

### Protein expression

Western blot analysis was performed as previously described (61). Briefly, cells were scraped, spun down, and stored at −80°C until analysis. Cells were thawed and resuspended in radioimmunoprecipitation assay buffer (50 mM Tris-HCl [pH 7.4], 150 mM NaCl, 1 mM EDTA, 1% NP-40, and 1% sodium deoxycholate) with cOmplete protease inhibitor cocktail (Roche). Samples were incubated on ice, spun at 4°C, and pellets discarded. Protein concentration was determined via Bradford assay. 6× loading dye (375 mM Tris-HCl [pH 6.8], 10% SDS, 30% glycerol, 0.6 M DTT, and 0.03% bromophenol blue) was added and samples boiled before loading equal protein amounts onto a 10% SDS-PAGE gel and transferred to nitrocellulose membranes (Amersham). Membranes were blocked in TBS-T (20 mM Tris-HCl [pH 7.6], 140 mM NaCl, and 0.1% Tween 20) with 5% milk (AmericanBio) at room temperature. Membranes were incubated with mouse monoclonal antibodies for 1 hour at room temperature or rabbit polyclonal antibodies at 4°C overnight. Membranes were washed with TBS-T and incubated with appropriate horseradish peroxidase (HRP) conjugated secondary antibodies in 1% bovine serum albumin (BSA) for 1 hour at room temperature and washed again with TBS-T before imaging. Western blots were developed by chemiluminescence using a digital imager (BioRad). Antibodies used were as follows: IE1 (1:1,000) (62), IE2 (1:500) (63), UL44 (1:1,000; Virusys), UL99 (1:1,000) (64), β-actin (1:500; SantaCruz), α-rabbit–HRP (1:10,000; SeraCare), and α-mouse–HRP (1:10,000; SeraCare). All experiments were performed in biological triplicate; representative Westerns were shown.

### Nucleic acid abundance

RNA abundance was analyzed as previously described (55, 65). Briefly, cell pellets were scraped and frozen at −80°C until analysis. Samples were thawed on ice and resuspended in TRIzol (Ambion) at room temperature. RNA was extracted with chloroform and pelleted with isopropanol. DNA was removed (TURBO DNase free kit, Ambion), and RNA was quantified via Nanodrop (ThermoScientific). Equal amounts of RNA were added to the reverse transcription reaction (High Capacity cDNA Reverse Transcription Kit, ThermoFisher). The thermocycler parameters were as follows: 25°C for 10 minutes, 37°C for 120 minutes, and 85°C for 5 minutes. RT-PCRs were performed using SYBR Green Select Master Mix (Applied Biosystems) and 0.5 µM gene-specific primers (Table 1). Comparison to a standard curve generated for each primer pair allowed for absolute quantification of gene product in samples. Viral genomes were extracted from equal volumes of virus stock for analysis and purified as previously described (55, 56, 59, 65). Briefly, DNA extraction buffer (400 nM NaCl, 10 mM Tris-HCl [pH 8.0], and 10 mM EDTA), 0.2% SDS, and 80 ng/mL proteinase K were added to samples, vortexed, and incubated overnight at 37°C. Samples were extracted with equal volumes of phenol/chloroform, treated with RNase A for 1 hour at 37°C and extracted with phenol/chloroform again. DNA was precipitated with ethanol and resuspended in 10 mM Tris-HCl (pH 8.0). Quantification of viral genomes was performed using qRT-PCR and UL99 primers. A standard curve of 10^1^–10^8^ copies was generated for UL99 to determine HCMV copy numbers from CT values.

### Polysome analysis

Analysis of mRNA translation efficiency (TE) was performed as previously described (55, 56, 65, 66). Briefly, MRC5 primary fibroblasts were infected with virus at a multiplicity of infection (MOI) of 3 for 1 hour, virus aspirated off, and fresh media were replaced for 24 hours. At the time of harvest, 100 mg/mL cycloheximide was added to the media for 10 minutes at 37°C. Cells were pelleted and resuspended in polysome lysis buffer (20 mM Tris-HCl [pH 7.4], 140 mM KCl, 5 mM MgCl_2_, 0.1% Triton X-100, and 10 mM DTT) and disrupted by passage through a 27 gauge needle 5 times. Nuclei were removed by centrifugation for 5 minutes at 2,500 rcf, and mitochondria were removed by centrifugation for 10 minutes at 13,000 rcf. Cytosolic lysate was loaded onto a 10%–50% linear sucrose gradient and spun via ultracentrifugation at 35,000 rpm for 2 hours without break. Gradients were fractionated and monitored by UV at 254 nm. RNA was extracted from equal volumes of polysome fractions as previously mentioned, and equal volumes were added into the cDNA reaction. TE was calculated by dividing the abundance of an mRNA transcript associated with polysomes (fractions 7–10) by the total abundance for that transcript across the gradient, normalized to wild-type (WT) virus TE.

### Viral growth assays

MRC5 primary fibroblasts were infected with an equal number of viral genomes equal to a WT multiplicity of infection of 3 for 1 hour at 37°C with rocking every 15 minutes. The virus was replaced with fresh media until time of harvest, and supernatants were stored at −80°C until analysis. The TCID_50_ method was used to quantify infectious units in the supernatant and cell-free viral genomes were quantified via qRT-PCR with primers specific for UL 99 (Table 1).

## RESULTS

### RNA secondary structure of the MIE1/2 5’ UTR

Multiple 5′ UTR features can impact mRNA TE, including length, sequence composition, and the presence of RNA secondary structure. The MIE 5′ UTR is 136 base pairs in length, similar to the average length of 5′ UTRs in human mRNAs (approximately 200 nucleotides [67]). Like the rest of the HCMV genome, the MIE 5′ UTR has a high GC content compared to the human genome. As elevated GC content generally favors the formation of stable RNA secondary structures, this suggests that the MIE 5′ UTR is likely to fold into RNA structures that may play a role in the regulation of IE1 and IE2 expression by the MIE 5′ UTR.

**Fig 1:**
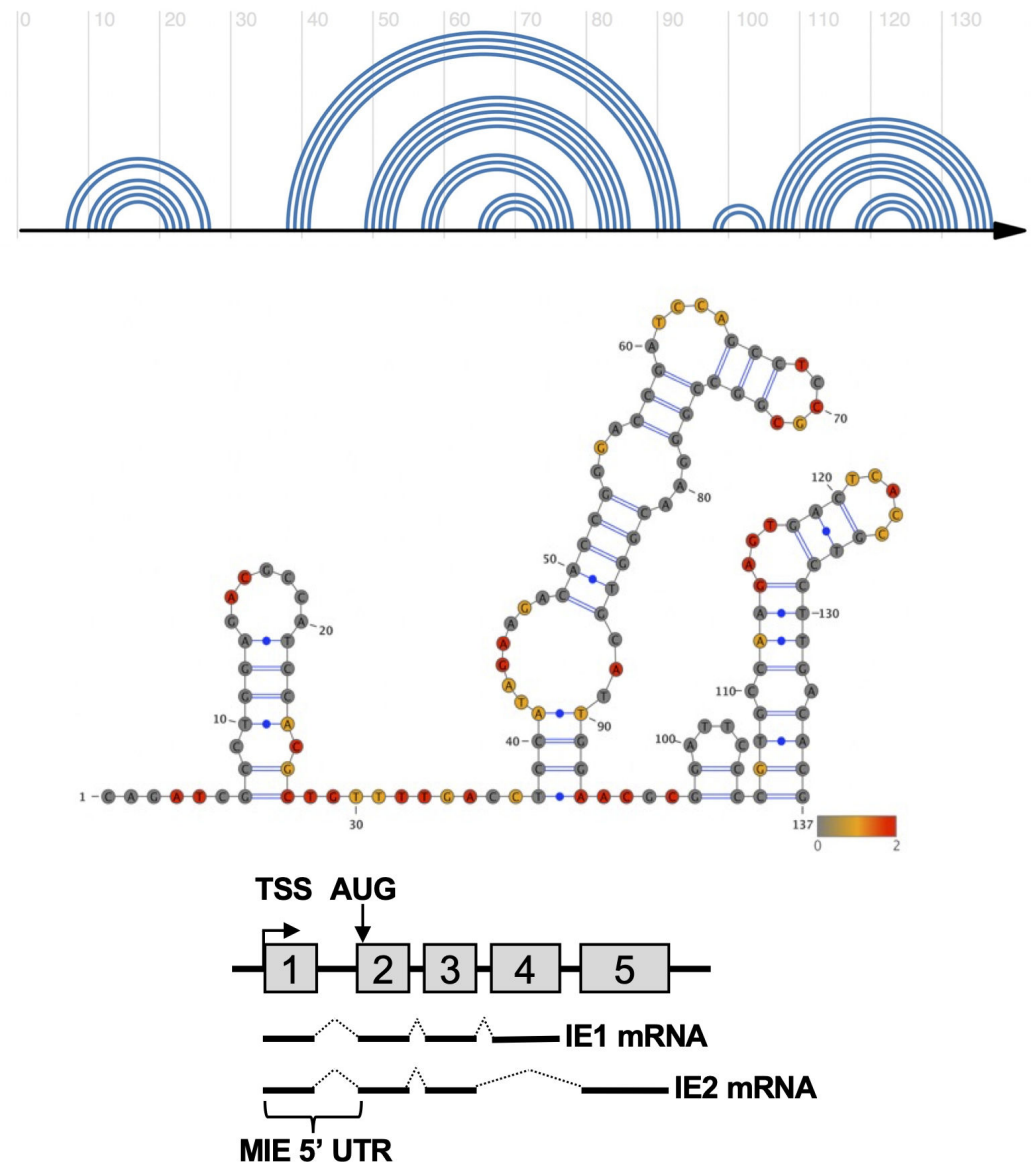
SHAPE-directed RNA structural model of the MIE 5′ UTR. SHAPE-Map analysis of the *in vitro* transcribed MIE 5’UTR was performed as described in Materials and Methods. (Top) Arch diagram representation of the MIE 5’ UTR RNA secondary structure. (Middle) Structural diagram of the MIE 5′ UTR with SHAPE reactivity overlaid. Nucleotide color indicates low (gray), medium (yellow), or high (red) SHAPE reactivity. (Bottom) Cartoon of the MIE genomic locus, indicating the location of the MIE 5′ UTR, transcription start site (TSS), translation start site (AUG), and structure of the mature IE1 and IE2 transcripts.

To determine if RNA secondary structure in the MIE 5′ UTR could regulate IE1 and IE2 expression, we used SHAPE-MaP to identify stable RNA secondary structures in the MIE 5′ UTR (Fig. 1). In SHAPE-MaP, an RNA is incubated with a chemical probe that covalently binds to unpaired nucleotides. During reverse transcription, the presence of an adduct results in mutations in the resulting cDNA, which are quantified at single nucleotide resolution using next-generation sequencing. The mutation profile is then combined with computational predictions of free energy and entropy to generate an experimentally validated model of RNA secondary structure.

**Fig 2:**
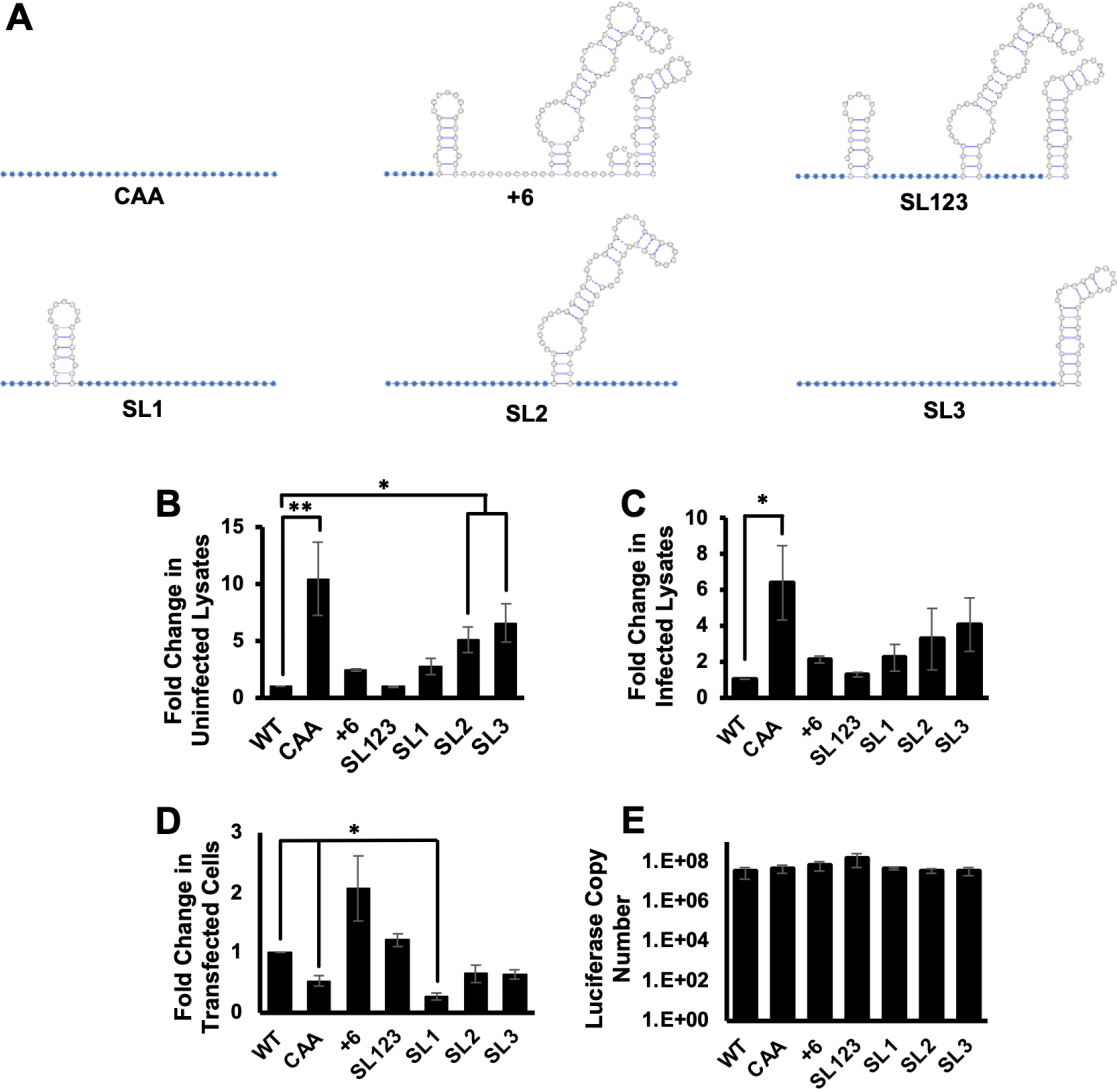
MIE 5′ UTR secondary structure inhibits translation *in vitro*. (**A**) Cartoon showing RNA secondary structure of MIE 5′ UTR mutants. Blue circles indicate nucleotides changed to CAA repeats; white nucleotides are unchanged from WT sequence. (**B and C**) Caped and polyadenylated *in vitro* transcribed RNAs containing the WT or mutant MIE 5′ UTR sequences upstream of the luciferase coding region were mixed with cytosolic extracts from uninfected (**B**) or infected (**C**) fibroblasts, and the fold change in luciferase activity was measured, with the activity of the reporter containing the WT MIE 5′ UTR set to 1. (**D**) MIE 5′ UTR luciferase transcripts were transfected into HeLa cells, and luciferase activity was measured. (**E**) The RNA abundance of each reporter in transfected cells was quantified using RT-qPCR. The graphs show the combined results of three independent experiments (*P < 0.05, **P < 0.01, multiple comparison statistics calculated using Dunnett’s test).

SHAPE-MaP analysis of the MIE 5′ UTR identified three stable stem loops (Fig. 1). Stem loop 1 (SL1) includes 21 nucleotides proximal to the 5′ cap, followed by a larger stem loop 2 (SL2) consisting of 60 nucleotides and a third stem loop of 32 nucleotides at the 3′ end of the MIE 5′ UTR (SL3). To determine how specific RNA structures in the MIE 5′ UTR impact translation, we constructed a series of reporter RNAs, each containing mutations in specific RNA structures or sequences (Fig. 2A). Cap-proximal nucleotides can play critical roles in translation initiation factor recruitment; therefore, we replaced the first six nucleotides of the MIE 5′ UTR with two CAA repeats (+6). We also generated a series of mutants that retain only a single stem loop, with all other nucleotides in the MIE 5′ UTR replaced with CAA repeats (SL1, SL2, and SL3). We also generated a reporter construct where all unpaired nucleotides between the three stem loops were replaced with CAA repeats (SL123). CAA repeats were used as they have previously been shown not to engage RNA secondary structure formation (56, 68, 69). To control for the impact of 5′ UTR length on mRNA TE, in each case, we maintained the wild type length of the 5′ UTR. As a control, we included the previously described reporter where all nucleotides in the MIE 5′ UTR were replaced with CAA repeats (56).

Each reporter gene was *in vitro* transcribed and then added to translation-competent cytosolic extracts derived from uninfected primary human fibroblasts (Fig. 2B). We found that outside the context of infection, the WT MIE 5′ UTR inhibited translation compared to the unstructured CAA control, similar to previous results (56). Replacing the first six nucleotides of the MIE 5′ UTR with CAA repeats did neither significantly impact translation as compared to the WT MIE 5′ UTR nor did replacing all unstructured nucleotides between stem-loops. The reporter which kept only SL1 intact was translated with a similar efficiency as the WT MIE 5′ UTR. In contrast, the presence of SL2 or SL3 alone increased reporter translation *in vitro* compared to the WT MIE 5′ UTR, with SL3 having a greater positive impact than SL2. Similar results were observed when the reporter transcripts were evaluated in translation-competent cytosolic extracts derived from HCMV-infected fibroblasts (Fig. 2C). As a further test, we measured the TE of each reporter transcript in transfected HeLa cells (Fig. 2D). Here, the lack of RNA sequence and structure with the CAA construct results in a reduction in luciferase activity compared to the WT construct. The differences in luciferase expression in HeLa cells were not due to differences in luciferase RNA levels (Fig. 2E). These data suggest that unstructured nucleotides adjacent to the 5’ mRNA cap or between stable RNA structures do not contribute to the regulation of reporter gene translation in cell-free extracts. The varying results in the different systems may suggest a context-dependent role for the MIE 5′ UTR in post-transcriptional control, or alternatively these reductionist systems each capture unique functions of the translational regulation. We provide these data in the context of the studies below to highlight the need to confirm results from more simplified systems in the more context of HCMV infection.

### Stem loop 1 is sufficient for HCMV replication

To determine the role of specific MIE 5′ UTR structures and sequences in IE1 and IE2 expression in the more relevant and complex environment of HCMV infection, we generated a series of recombinant viruses encoding the MIE 5′ UTR mutants shown in Fig. 2A. A virus where all nucleotides in the MIE 5′ UTR were replaced with CAA repeats served as a control (56). Two independent isolates of each virus were tested in all experiments to ensure that any observed phenotypes were due to the intended mutations rather than off-target effects.

**Fig 3:**
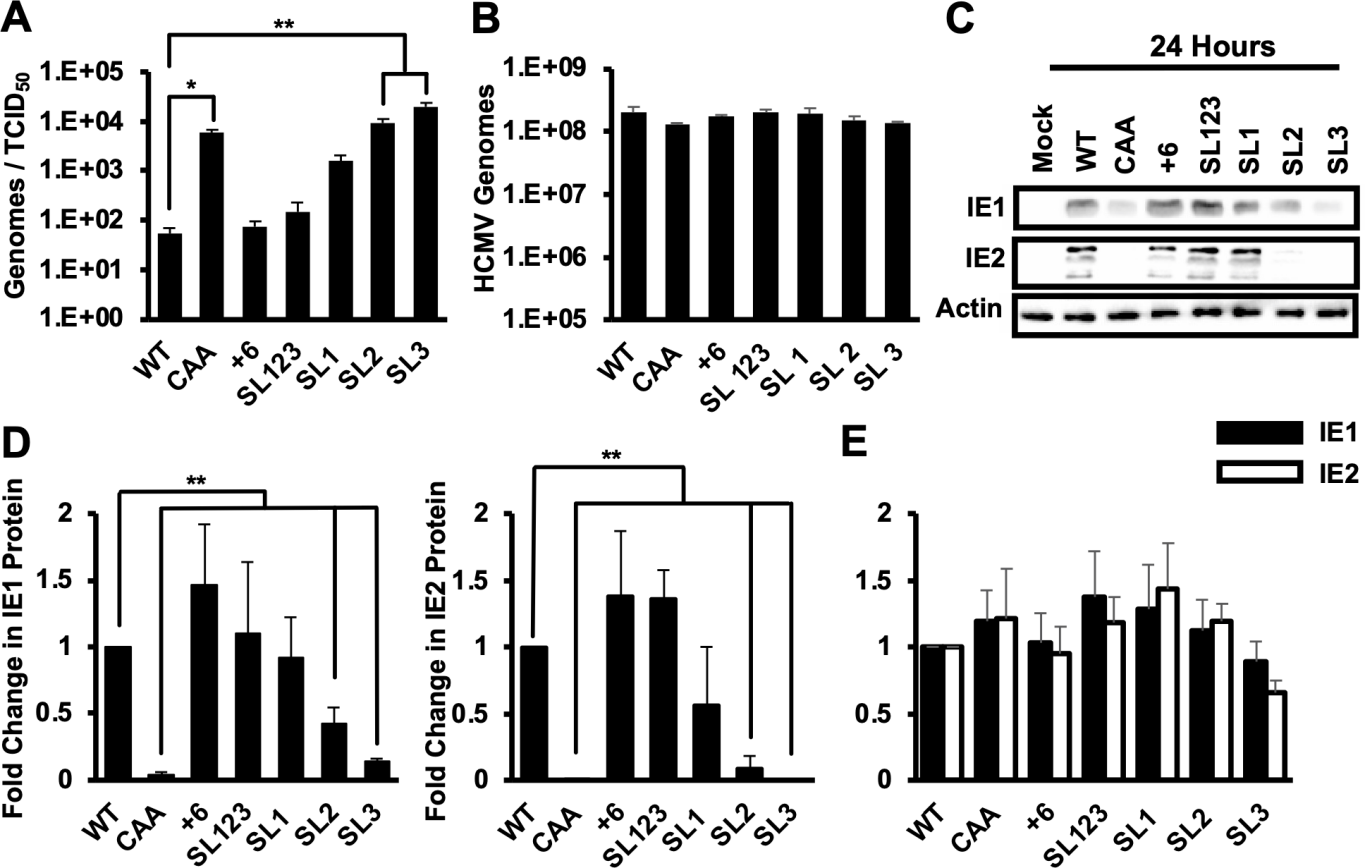
Stem loop 1 is sufficient for IE1 and IE2 expression early in infection. (**A**) The number of HCMV genomes and infectious virus particles was determined by RT-qPCR and the TCID_50_ assay, respectively. The graph shows the ratio of HCMV genomes to infectious particles. (**B**) MRC5 primary fibroblasts were infected with an equivalent number of genomes of each virus, which was equivalent to the number of genomes present after infection with wild type virus at a multiplicity of infection of 3. (**C**) Protein expression was analyzed via Western blot at 24 hours post infection for IE1, IE2, or the β-actin loading control. The results representative of three independent experiments. (**D**) Quantification of Western blots at 24 hours post infection in (**C**). SEM for three independent experiments for fold change in IE1 (left) or IE2 (right) protein levels is compared to WT infected cells, which is set to 1. (**E**) Cells were infected as in (**C**), and the abundance of transcripts encoding either IE1 (black bars) or IE2 (white bars) was measured at 24 hours after infection by RT-qPCR. The fold change in RNA levels compared to cells infected with WT virus is shown. The data show the average of three independent experiments. All experiments confirmed with two independent clones of each recombinant virus (*P < 0.05, **P < 0.01, multiple comparison statistics calculated using Dunnett’s test).

We previously found that replacing the MIE 5′ UTR with CAA repeats resulted in an increase in the particle to infectious virus ratio (56). To determine if any of the MIE 5′ UTR mutations has a similar effect, we measured the particle to infectious virus ratio in the viral stocks of each of our mutants (Fig. 3A). We found that the CAA, SL2, and SL3 viruses each had an approximately 100-fold increase in the particle to infectious virus ratio compared to WT virus. The SL1 virus had a 10-fold increase, while the +6 and SL123 viruses did not have a significant difference in particle to infectious virus ratio as compared to WT virus stocks. The defect in particle to infectious virus ratio for the CAA, SL1, SL2, and SL three viruses is not due to defects in virion formation or infectivity, as there were similar numbers of intracellular viral genomes after infection with an equivalent number of viral genomes for each virus (Fig. 3B). To control for potential differences resulting from infecting with different numbers of viral genomes, subsequent experiments were performed using infection with an equivalent number of viral genomes for each virus, which was equal to the number of genomes present after infection with wild type virus at a multiplicity of 3 infectious units per cell.

To determine how each 5′ UTR mutation impacted IE1 and IE2 expression, we measured IE1 and IE2 RNA and protein levels in infected cells at 24 hours after infection by qRT-PCR and Western blot, respectively. IE1 and IE2 protein levels were decreased in cells infected with the SL2 and SL3 viruses as compared to wild type virus infection (Fig. 3C and D). The recombinant viruses SL1, SL123, and +6 each expressed both IE1 and IE2 protein to similar levels as wild type. There was no statistical difference in IE1 or IE2 mRNA abundance at 24 hours post infection for any of the recombinant viruses as compared to wild type infection (Fig. 3E).

**Fig 4:**
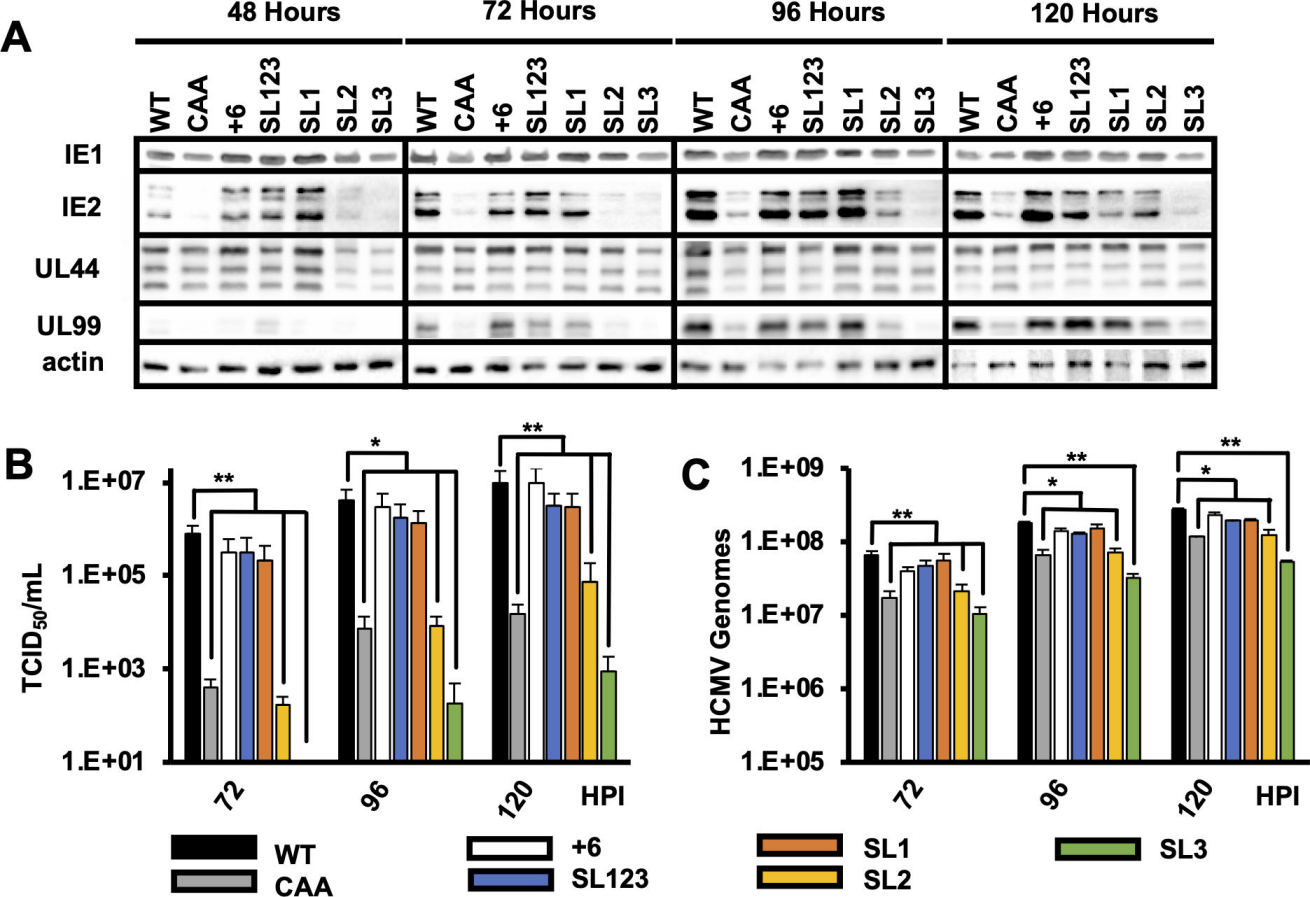
Disrupting stem loop 1 decreases HCMV replication. (**A**) MRC5 fibroblasts were infected with equivalent number so viral genomes for each mutant as in Fig. 3. Viral protein expression for representative immediate early (IE1 and IE2), early (UL44), and late (UL99) proteins was measured by Western blot at the indicated times after infection. Representative results from three independent experiments are shown. (**B**) Cell-free virus was measured at 72, 96, and 120 hours after infection with each virus using the TCID_50_ method. (**C**) Cell-free HCMV genomes were quantified at the indicated times after infection by qPCR. Results show the SEM for three independent experiments. Statistical significance was determined by pairwise comparison to WT values. All experiments were confirmed with two independent clones of each recombinant virus (*P < 0.05, **P < 0.01, multiple comparison statistics calculated using Dunnett’s test).

To determine the role of RNA structure on HCMV replication, we measured the expression of representative immediate early (IE1 and IE2), early (UL44), and late (UL99) viral proteins over a single round of virus replication. Immediate early, early, and late proteins were expressed to similar levels with equivalent kinetics after infection with viruses SL1, SL123, and +6 as compared to wild type virus. In contrast, there was delayed and reduced IE1 and IE2 expression and decreased UL44 and UL99 expression after infection with the SL2 and SL3 viruses (Fig. 4A). Fewer cell free infectious virions (Fig. 4B) and viral genomes (Fig. 4C) were produced after infection with the SL2 and SL3 viruses, consistent with the defect in viral protein expression. No significant difference was found in viral genome accumulation or cell-free virus levels after infection with the +6, SL123, and SL1 viruses. These data suggest that the cap-proximal nucleotides and nucleotides between the stem loops of the MIE 5′ UTR are not essential for efficient HCMV replication. Moreover, these data show that SL1 of the MIE 5′ UTR is sufficient for HCMV replication.

### Stem loop 1 is necessary for efficient translation of IE1 and IE2 mRNA translation

**Fig 5:**
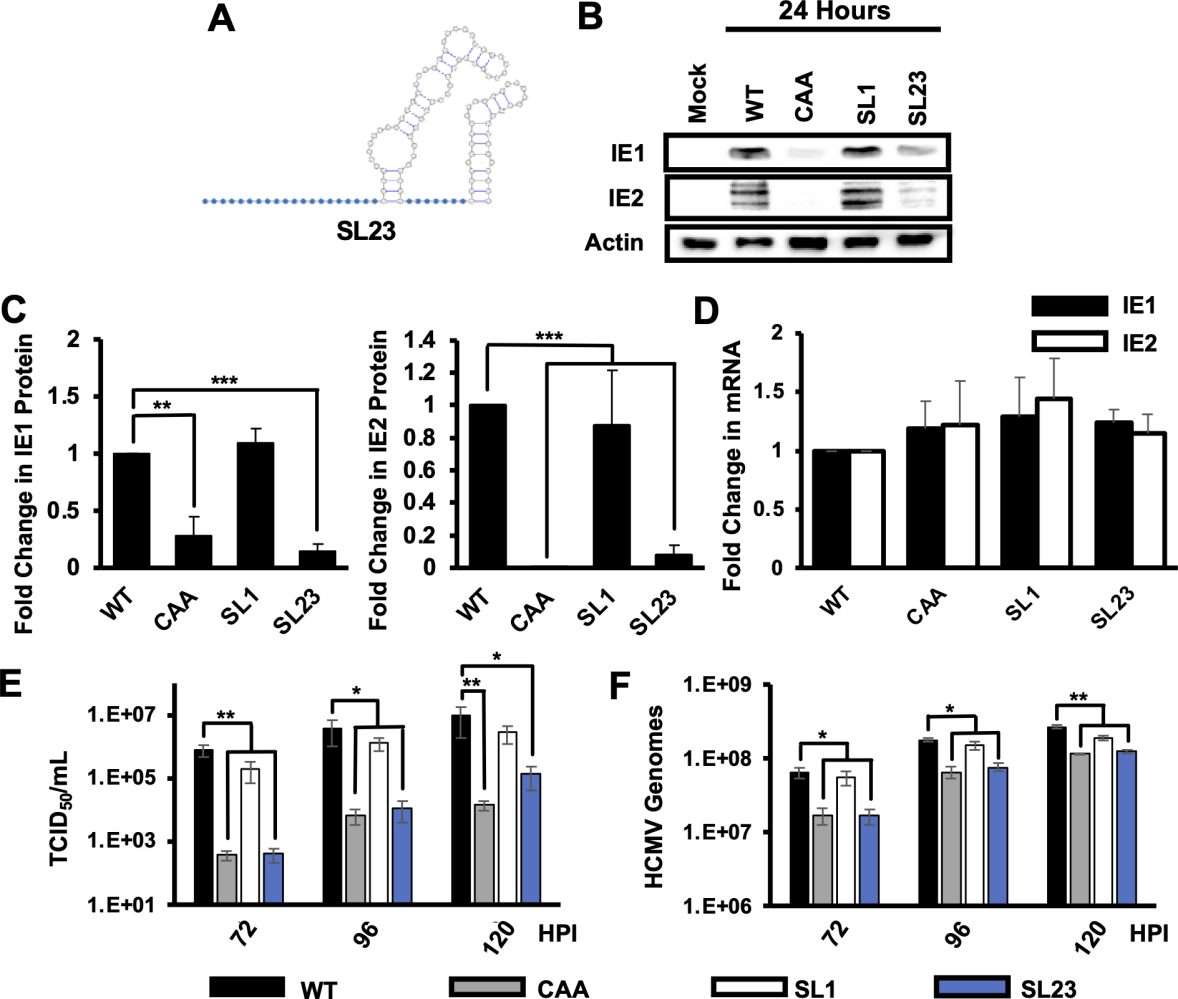
Stem loop 1 is necessary for efficient IE1 and IE2 expression. (**A**) Cartoon of SL23 MIE 5’ UTR. (**B**) Protein levels for IE1 and IE2 were measured by Western blot at 24 hours after infection. Representative results are shown. (**C**) Quantification of relative IE1 (left) or IE2 (right) protein expression from three independent experiments. Protein levels in WT infected cells are set to 1. (**D**) Fold change in the abundance of transcripts encoding either IE1 (black) and IE2 (white) at 24 hours after infection as determined by RT-qPCR values in cells infected with WT virus were set to 1. Cell-free infectious virus (**E**) or HCMV genomes (**F**) were measured by the TCID_50_ method or qPCR, respectively, at the indicated times after infection. The graphs show the average and SEM for three independent experiments. All experiments were confirmed with two independent clones of each recombinant virus. Statistical significance was determined by pairwise comparison to values from cells infected with WT virus (*P < 0.05, **P < 0.01, multiple comparison statistics calculated using Dunnett’s test).

We next determined if SL1 of the MIE 5′ UTR was necessary for efficient HCMV protein expression and virus replication. We generated a recombinant virus where SL1 was removed, but SL2 and SL3 remained intact (SL23; Fig. 5A). The SL23 virus was as infectious as wild type virus, as intracellular viral DNA levels were similar to those in wild type infected cells at 6 hours after infection (not shown). We observed decreased IE1 and IE2 proteins levels after SL23 infection as compared to wild type virus (Fig. 5B and C), though IE1 and IE2 mRNA levels were comparable (Fig. 5D). The SL23 virus also produced significantly less cell free infectious virus as compared to wild type virus (Fig. 5E and F). Together with the data in Fig. 4, these data show that SL1 is necessary and sufficient for efficient IE1 and IE2 expression and HCMV replication.

**Fig 6:**
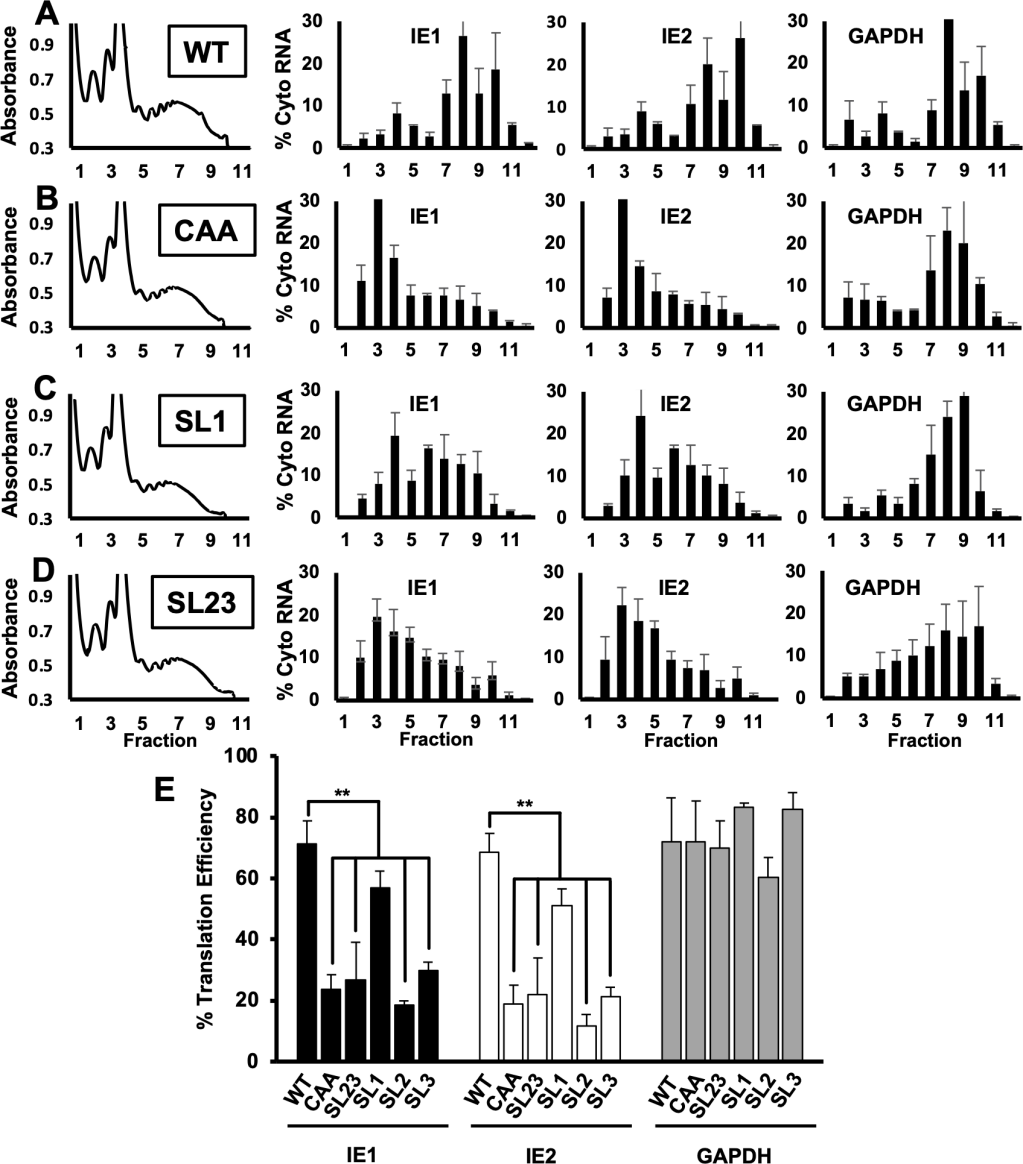
Stem loop one is required for efficient translation of IE1 and IE2. MRC5 fibroblasts were infected with as in Fig. 3 with equivalent numbers of viral genomes of (**A**) WT, (**B**) CAA, (**C**) SL1, or (**D**) SL23 virus. At 24 hours after infection, cytosolic lysates from infected cells were resolved through a 10%–50% linear sucrose gradient. The abundance of RNAs encoding IE1, IE2, or GAPDH in gradient fraction was measured by RT-qPCR. The graphs show the percent of each RNA in each gradient fraction compared to the total amount of each RNA in all gradient fractions combined. (**E**) The translation efficiency of RNAs encoding IE1 (black bars), IE2 (white bars), or GAPDH (gray bars) was determined by dividing the abundance of each RNA in fractions containing polysomes (fractions 6–10) and by the total abundance of each RNA. Data shown are the SEM for three independent experiments; statistical significance was determined by pairwise comparison to WT values. Experiments confirmed with two independent clones of each recombinant virus (**P < 0.01, multiple comparison statistics calculated using Dunnett’s test).

The decrease in IE1 and IE2 protein levels despite wild type levels of IE1 and IE2 mRNA in the absence of SL1 suggested that SL1 may serve to stimulate translation of the IE1 and IE2 mRNA. To more precisely examine the role of SL1 in the translation of the IE1 and IE2 mRNA, we used sucrose gradient density centrifugation to measure the association of the IE1 and IE2 mRNAs with polysomes after infection with wild type virus or the SL1 or SL23 viruses. The CAA virus served as a control. The overall abundance of polysomes in infected cells was similar after infection with each virus, and the distribution of GAPDH mRNA across the gradient was similar for all viruses (Fig. 6A through D, right panels), indicating that the mutations did not affect the overall level of translation in infected cells (Fig. 6A through D, left panels). For the wild type virus, the IE1 and IE2 mRNAs were most abundant in gradient fractions containing polysomes (fractions 6–10; Fig. 6A), indicating that the IE1 and IE2 mRNAs are efficiently translated. After infection with the CAA control (Fig. 6B), less IE1 and IE2 mRNA were found in fractions containing polysomes, with a concomitant increase in IE1 and IE2 mRNA levels in gradient fractions containing monosomes (fractions 3–5), suggesting a reduction in TE, consistent with previous results (56). IE1 and IE2 mRNA was found in monosome and polysome-containing fractions after infection with the SL1 virus (Fig. 6C), whereas IE1 and IE2 transcripts predominantly accumulated in gradient fractions containing monosomes after infection with the SL23 virus, similar to the CAA control virus (Fig. 6D).

To better quantify the impact of each MIE 5’ UTR mutation on IE1 and IE2 mRNA translation, we determined the TE for each transcript after infection with each virus by dividing the abundance of each transcript gradient fractions that contained polysomes by the total abundance of the transcript in the entire gradient. The TE of the IE1 and IE2 mRNAs was 71.3% ± 7.6% and 68.7% ± 6.0%, respectively, after infection with wild type virus (Fig. 6E). As previously reported (56), there was a statistically significant decrease in the TE of both the IE1 and IE2 mRNAs (23.6% ± 4.9% and 18.8% ± 6.2%, respectively) after infection with the CAA control virus. A decrease in the TE for both IE1 and IE2 mRNAs was also observed after infection with the SL2, SL3, and SL23 viruses as compared to wild type virus. A slight decrease in the TE for both IE1 and IE2 was seen after infection with the SL1 virus (57.0% ± 5.3% and 51.1% ± 5.4% respectively), though this difference was not statistically significant. No difference in the TE of the GAPDH mRNA was found after infection with the mutant viruses as compared to wild type virus. These data support the conclusion that SL1 in the MIE 5′ UTR is critical for the efficient translation of IE1 and IE2 mRNA during HCMV infection.

### The structure and location of SL1 is critical for efficient IE1/2 expression and HCMV replication

**Fig 7:**
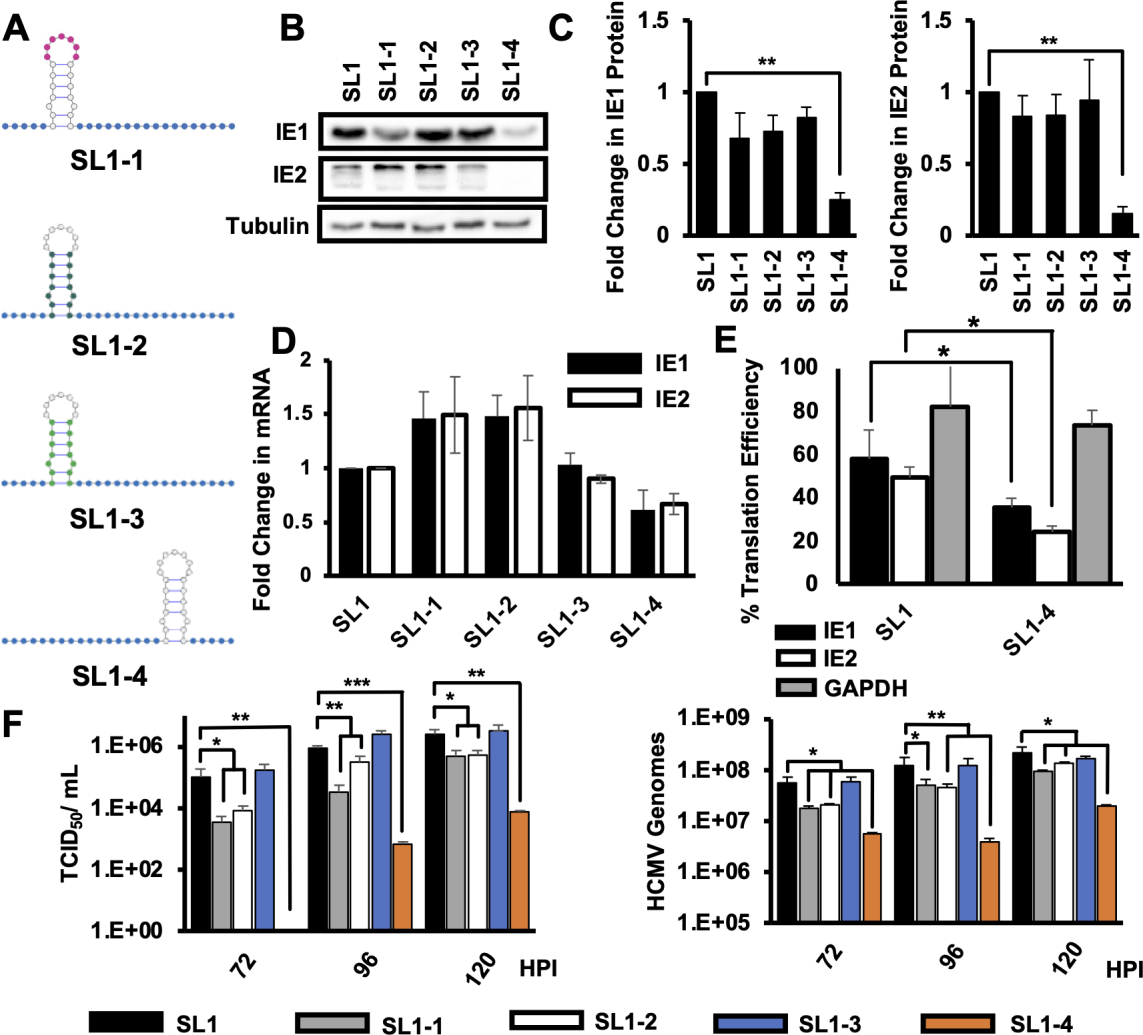
The location of SL1 is critical for efficient IE1 and IE2 expression and HCMV replication. (**A**) Cartoon showing mutations impacting different SL1 features. Blue nucleotides were replaced with to CAAs repeats. Pink nucleotides were changed to AAAAAAA, while dark green nucleotides have been shuffled from the WT sequence but leave the structure intact. Light green nucleotides were changed to a synthetic stem loop with the same ΔG as the WT SL1, and uncolored nucleotides were unchanged from the WT sequence. (**B**) MRC5 fibroblasts were infected as in Fig. 3, and IE1, IE2, or tubulin protein levels were measured by Western blot at 24 hours after infection. A representative of three independent experiments is shown. (**C**) Quantification of Western blot data at 24 hours after infection as in panel **B**. The average and SEM for three independent experiments is shown, with values from WT infected cells set to 1. (**D**) Fold change in IE1 (black bars) or IE2 (white bars) RNA levels compared to cells infected with WT virus was determined by RT-qPCR at 24 hours after infection, with the value in WT cells set to 1. (**E**) The TE for transcripts encoding IE1 (black), IE2 (white), or GAPDH (gray) was determined at 24 hours after infection as in Fig. 5. (**F**) Cells were infected as above, and the number of infectious virus particles (left panel) or HCMV genomes (right panel) in the supernatant was determined at 72, 96, or 120 hours post infection. The graphs show the average of three independent experiments, and all experiments confirmed with two independent clones of each recombinant virus. Statistical significance was determined by pairwise comparison to infection with the SL1 virus. (*P < 0.05, **P < 0.01, multiple comparison statistics calculated using Dunnett’s test, student’s *t* test was used in pairwise comparisons).

We next made a series of recombinant viruses to define specific features of SL1 required for efficient IE1 and IE2 expression (Fig. 7A). Single-stranded loops in stem-loop structures can serve as binding sites for RNA-binding proteins that regulate translation. We, therefore, made a recombinant virus where the seven nucleotides in the wild type sequence SL1 loop sequence (GACGCCA) were changed to AAAAAAA (SL1-1). We made two additional recombinants where the nucleotides of the stem sequence were shuffled while keeping the structure intact (SL1-2), or the entirety of SL1 was replaced with a synthetic stem-loop with the same ΔG (SL1-3) to measure the potential role for sequence-specific double-stranded RNA binding proteins in SL1 function. We also made a recombinant where SL1 was moved further 3′ from the 5′ cap to determine the effect of SL1 position on its ability to enhance IE1 and IE2 mRNA translation (SL1-4, Fig. 7A). We then infected human fibroblasts with an equivalent number of viral genomes of each virus as before and determined the effect on IE1/2 protein expression at 24 hours after infection. As before, none of the mutations impacted infectivity (not shown). Changing the sequence of the SL1 loop, shuffling the nucleotides of the SL1 stem, or replacing SL1 with a synthetic stem-loop did not affect IE1 or IE2 protein or RNA expression as compared to the SL1 virus (Fig. 7B through D). However, moving SL1 further from 3′ from the 5′ mRNA cap significantly decreased both IE1 and IE2 protein levels as compared to SL1, while IE1 and IE2 mRNA levels were unaffected (Fig. 7B through D). We found that both the IE1 and IE2 mRNAs were translated less efficiently when SL1 was moved (35.6% ± 4.1% and 24.0% ± 3.0% TE compared to WT, respectively) compared to SL1 virus (57.8% ± 13.5% and 49.4% ± 5.0% TE, respectively, Fig. 7E). while the TE of GAPDH mRNA was not affected. Replacing the loop sequence or shuffling the stem did not affect the production of infectious particles or viral genomes (Fig. 7F), while moving SL1 farther from the 5′ cap resulted in a significant decrease in both infectious virion production and viral DNA accumulation. From this, we conclude that the presence of RNA structure at the 5′ end of the IE1 and IE2 mRNAs is necessary for efficient IE1 and IE2 expression and HCMV replication.

## DISCUSSION

The 5′ UTR of an mRNA can moderate protein expression at the level of translation. Our results show that RNA secondary structure of the MIE 5′ UTR enhances IE1 and IE2 expression in the context of HCMV infection. We found that the MIE 5′ UTR contains three stable stem loop structures. Within the context of infection, disruption of this secondary structure increases the particle to infectious unit ratio without altering HCMV entry into the cell. We speculate that the difference in particle to infectious virus ratio we observe when propagating virus is the result decreased IE1 and IE2 protein expression during the immediate early stage of infection, where they play critical roles in establishing lytic infection and activating subsequent early gene expression. These data are similar to the results seen after infection with and IE1 deletion virus at low multiplicity of infection (70, 71). Additionally, disruption of the MIE 5′ UTR structure does not impact the transcription of the IE1 or IE2 mRNAs, yet some mutations result in decreased IE1 and IE2 protein expression, delayed early and late gene expression, and reduced HCMV replication. Our work highlights the importance of RNA secondary structure in the regulation of IE1 and IE2 expression and HCMV replication.

We first studied the impact of the MIE 5′ UTR on post-transcriptional regulation in simplified systems using *in vitro* transcribed RNA mixed with translation-competent cytosolic extracts or transfected into a standard tissue culture cell line. As we previously observed (56), the results in these systems differ from those seen in HCMV-infected cells. These differences may reflect context-specific roles for the MIE 5′ UTR in different settings. Alternatively, these differences could reflect the fact that infection induces changes to cellular signaling pathways and gene expression that impact the function of the MIE 5′ UTR. We include these results to highlight the need to consider the potential limitations of *in vitro* systems and the necessity of confirming the results from such systems in the more complex and relevant environment of HCMV infection.

RNA structure in the 5′ UTR can have opposing effects on translation for different mRNAs. In some cases, RNA structure impedes ribosome scanning or recognition of the translation start site (46–50) to decrease translation. In others, specific RNA structures act to recruit factors that enhance ribosome loading to increase TE (45). Our results show that SL1 in the MIE 5′ UTR plays a critical role in HCMV replication by enhancing the translation of mRNAs encoding IE1 or IE2; SL1 is both necessary and sufficient for IE1 and IE2 mRNA translation in the context of HCMV infection. However, the mechanism by which SL1 enhances translation remains unknown. Our unpublished data suggest that the MIE 5′ UTR does not act as an internal ribosome entry site, suggesting a role in recruiting factors that increase cap-dependent translation. Surprisingly the increase in TE was independent of the specific sequence of the SL1 stem. This suggests that the presence of RNA structure, rather than a specific nucleic acid sequence, serves as the recognition site for factors that enhance ribosome recruitment, though additional studies are needed to test this hypothesis. Perhaps SL1 mediates interactions between the 5′ UTR and 3′ UTR, or between specific factors bound to each, to stabilize translation initiation complex formation and processing. In any case, efforts to identify factors that bind the MIE 5′ UTR, and SL1 in particular, are likely to reveal critical factors in the control of IE1 and IE2 expression.

Our finding that the MIE 5′ UTR is a critical regulator of IE1 and IE2 expression may also have implications in other infection settings. HCMV infects a wide range of cell types, each with its own unique complement of RNA-binding proteins. These differences could result in changes in IE1 and IE2 expression in different cell types, contributing to changes in HCMV replication efficiency or kinetics. Repressive factors could bind the MIE 5′ UTR in specific cell types to decrease IE1 and IE2 expression leading to the establishment of latency. Conversely, differentiation of latently infected cells might change the expression of factors that enhance translation of the mRNAs encoding IE1 and IE2 to drive reactivation. This may involve interactions with SL1, though other RNA structures or sequences in the MIE 5′ UTR may have specific roles in specific infection settings that were not captured in our studies in lytically infected fibroblasts. Defining the role of each RNA structure and associated factors in different cell types may therefore reveal additional mechanisms regulating key decision points in the HCMV replicative cycle.

Our studies here focus on the 5′ UTR of mRNAs originating from the MIEP. In other work, we found that alternative promoters in the MIE locus regulate the expression of mRNAs encoding full-length IE1 and IE2 proteins in other cell types (55, 72). The most obvious impact of promoter switching is the change in factors controlling transcription. However, it is important to note promoter switching can also lead to changes to the RNA structure and sequence of the 5′ UTR, coupling changes in transcription control to changes in the control of mRNA translation. MIE transcripts arising from these alternative promoters differ widely in the length of their 5′ UTR, and *in silico* predictions suggest significant changes to RNA structure in the 5′ UTR. Together with the changes in transcriptional control, these 5′ UTR changes could provide for finely tuned IE1 and IE2 expression in response to different cellular or environmental cues.

Our results with the MIE 5′ UTR raise the possibility that other viral 5′ UTRs to have similar effects in HCMV protein expression. Due to the complexity of the HCMV genome and the potential for cell-type-specific regulation, we still lack a complete catalog of HCMV transcripts and thus a full understanding of viral 5′ UTR sequences. In addition, some HCMV genes are encoded on multiple transcripts that differ in their 5′ UTR and temporal expression profile (55, 73–76), suggesting that temporal changes in 5′ UTR usage, and thus RNA secondary structure, may be important for regulating HCMV gene expression during specific stages of the virus lifecycle or in specific cell types. A more complete knowledge of viral 5′ UTR sequence and structure is therefore likely to illuminate additional regulatory events controlling HCMV protein expression.
